# Inhibition of PRMT1 Suppresses the Growth of U87MG-Derived Glioblastoma Stem Cells by Blocking the STAT3 Signaling Pathway

**DOI:** 10.3390/ijms25052950

**Published:** 2024-03-03

**Authors:** Nayeong Yuk, Hye Jin Jung

**Affiliations:** 1 Department of Life Science and Biochemical Engineering, Graduate School, Sun Moon University, Asan 31460, Republic of Korea; nayeong7249@sunmoon.ac.kr; 2 Department of Pharmaceutical Engineering and Biotechnology, Sun Moon University, Asan 31460, Republic of Korea; 3 Genome-Based BioIT Convergence Institute, Sun Moon University, Asan 31460, Republic of Korea

**Keywords:** glioblastoma stem cell, protein arginine methyltransferase 1, furamidine, signal transducer and activator of transcription 3, calcium/calmodulin-dependent protein kinase II gamma, berbamine

## Abstract

Glioblastoma stem cells (GSCs) play a pivotal role in the initiation, progression, resistance to treatment, and relapse of glioblastoma multiforme (GBM). Thus, identifying potential therapeutic targets and drugs that interfere with the growth of GSCs may contribute to improved treatment outcomes for GBM. In this study, we first demonstrated the functional role of protein arginine methyltransferase 1 (PRMT1) in GSC growth. Furamidine, a PRMT1 inhibitor, effectively inhibited the proliferation and tumorsphere formation of U87MG-derived GSCs by inducing cell cycle arrest at the G0/G1 phase and promoting the intrinsic apoptotic pathway. Moreover, furamidine potently suppressed the in vivo tumor growth of U87MG GSCs in a chick embryo chorioallantoic membrane model. In particular, the inhibitory effect of furamidine on U87MG GSC growth was associated with the downregulation of signal transducer and activator of transcription 3 (STAT3) and key GSC markers, including CD133, Sox2, Oct4, Nanog, aldehyde dehydrogenase 1, and integrin α6. Our results also showed that the knockdown of PRMT1 by small interfering RNA significantly inhibited the proliferation of U87MG GSCs in vitro and in vivo through a molecular mechanism similar to furamidine. In addition, combined treatment with furamidine and berbamine, a calcium/calmodulin-dependent protein kinase II gamma (CaMKIIγ) inhibitor, inhibited the growth of U87MG GSCs more strongly than single-compound treatment. The increased antiproliferative effect of combining the two compounds resulted from a stronger downregulation of STAT3-mediated downstream GBM stemness regulators through dual PRMT1 and CaMKIIγ function blockade. In conclusion, these findings suggest that PRMT1 and its inhibitor, furamidine, are potential novel therapeutic targets and drug candidates for effectively suppressing GSC growth.

## 1. Introduction

Glioblastoma multiforme (GBM) is the most common primary malignant brain tumor in humans. Despite aggressive multimodal standard treatment approaches, GBM remains a fatal disease with a median survival of 15 months and a 5-year survival rate of less than 5% [1,2]. Temozolomide (TMZ) and bevacizumab, which are approved for GBM treatment, have serious clinical efficacy and safety limitations [3,4]. Therefore, there is an urgent need to explore more effective GBM treatments. Accumulating evidence has shown that glioblastoma stem cells (GSCs) are major contributors to the poor prognosis of GBM patients [5]. GSCs play important roles in tumor initiation, cancer invasion, immune evasion, therapeutic resistance to radiation and chemotherapy, and tumor recurrence [6,7]. Several critical regulators of GSC growth and maintenance have been identified. These include cell surface molecules such as CD133 and integrin α6; transcription factors such as Sox2, Oct4, and Nanog; cytosolic enzymes such as aldehyde dehydrogenase 1 (ALDH1); and signaling regulators such as Wnt/β-catenin, Notch, hedgehog, Janus kinase (JAK)/signal transducer and activator of transcription (STAT), and receptor tyrosine kinases [8,9]. Therefore, identifying potential molecular targets that promote the aberrant expression or activation of these GSC regulators may contribute to developing novel GSC-targeted therapies to improve the treatment and prognosis of GBM.

Protein arginine methyltransferases (PRMTs) catalyze the transfer of methyl groups to arginine residues in substrate proteins and are classified into three types (I, II, and III) depending on the type of methylarginine produced [10]. By methylating histones and a wide range of non-histone proteins, PRMTs are involved in diverse biological functions such as transcriptional regulation, DNA repair, signal transduction, and protein-protein interactions [11]. PRMT1, belonging to type I, mediates the formation of monomethylated arginine and asymmetric dimethylated arginine and is responsible for more than 85% of PRMT activity in mammalian cells [12]. Recent studies have demonstrated that the dysregulation of PRMT1 is closely implicated in cancer initiation, progression, and poor prognosis in patients with cancer [13]. PRMT1 is overexpressed in various cancer types, including GBM, and the upregulation of PRMT1 promotes cancer cell proliferation and metastasis by methylating several target proteins such as epidermal growth factor receptor (EGFR), GLI1, Twist1, enhancer of zeste homolog 2 (EZH2), cyclin-dependent kinase 4 (CDK4), and STAT [12]. In addition, the inhibition of PRMT1 using specific small-molecule inhibitors or gene silencing results in cell cycle arrest, proliferation inhibition, and apoptosis induction in several cancer cells, including glioma cells [13,14]. It has also been reported that PRMT1 inhibition in neural stem cells (NSCs) downregulates the survival, proliferation, and differentiation of NSCs [15,16]. Therefore, PRMT1 is recognized as a potential prognostic biomarker and therapeutic target in cancer. However, the functional role of PRMT1 in the growth and maintenance of cancer stem cells, including GSCs, remains unclear.

In this study, we assessed the inhibitory effects of furamidine, a specific PRMT1 inhibitor, and PRMT1-specific small interfering RNA (siRNA) on the growth of U87MG-derived GSCs in vitro and in vivo. Furthermore, we investigated whether the inhibition of PRMT1 by furamidine and siRNA affected STAT3 activation and key GBM stemness regulators in U87MG-derived GSCs. We also demonstrated an enhanced inhibitory effect by combined treatment of furamidine and berbamine, a calcium/calmodulin-dependent protein kinase II gamma (CaMKIIγ) inhibitor, on the growth of U87MG-derived GSCs. Thus, these findings suggest that PRMT1 plays a key role in GSC growth by upregulating STAT3-mediated downstream GBM stemness regulators and that the PRMT1 inhibitor furamidine, either alone or in combination with the CaMKIIγ inhibitor berbamine, may be a novel anticancer agent to eliminate GSCs.

## 2. Results

### 2.1. Furamidine Inhibits the Proliferation of U87MG-Derived GSCs Both In Vitro and In Vivo

**Figure 1:**
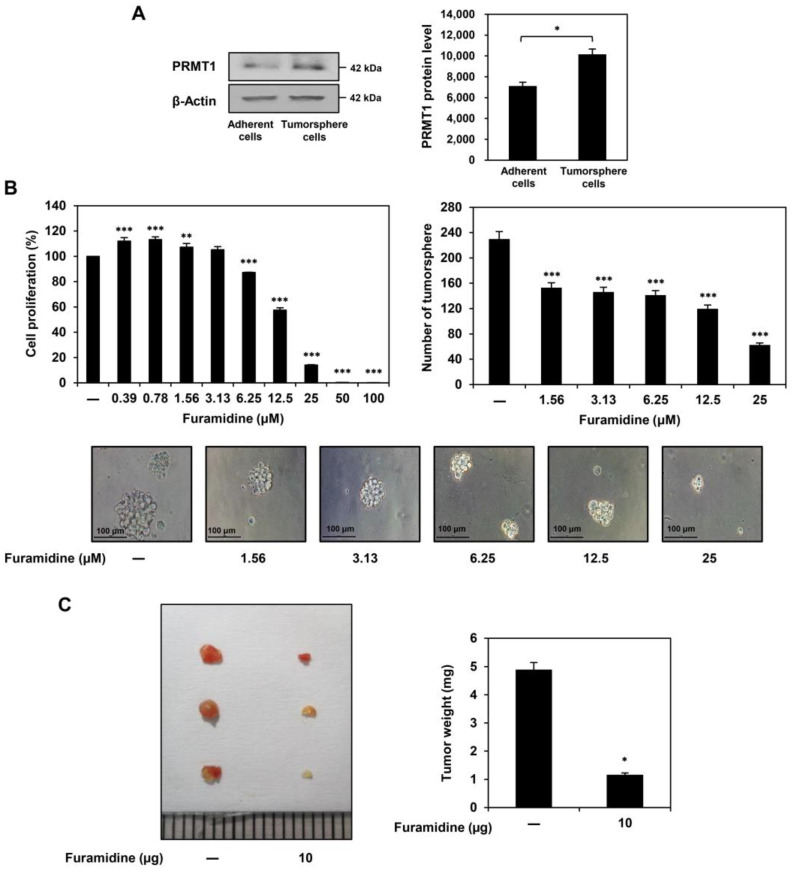
Effect of furamidine on proliferation of U87MG-derived GSCs in vitro and in vivo. (**A**) Protein expression levels of PRMT1 in U87MG adherent and tumorsphere cells. Protein levels were detected by Western blotting and quantified by densitometry. β-Actin levels were used as a loading control. * *p* < 0.05. (**B**) Effect of furamidine on the proliferation and tumorsphere formation in U87MG-derived GSCs. Cells were treated with furamidine at various concentrations (0–100 μM) for 7 days. Cell proliferation was measured using the CellTiter-Glo^®^ luminescent assay system. Formed tumorspheres were counted under an optical microscope. ** *p* < 0.005, *** *p* < 0.001 vs. the control. (**C**) Effect of furamidine on tumor growth derived by U87MG GSCs in a CAM model. U87MG-derived GSCs were mixed with ECM gel in the absence or presence of the furamidine (10 µg/egg) and placed onto the CAM surface of fertilized chick eggs. After incubation for 7 days, the size and weight of the formed tumors were calculated. * *p* < 0.05 vs. the control.

As previously reported, GSC populations were selectively propagated from the U87MG GBM cell line using serum-free spheroid suspension culture medium containing epidermal growth factor (EGF) and basic fibroblast growth factor (bFGF) [17,18]. The U87MG-derived GSCs significantly overexpressed key stemness markers, including CD133, Sox2, Oct4, Nanog, ALDH1A1, and integrin α6, compared to those of non-stem U87MG adherent cells (Figure S1). PRMT1 protein expression levels were higher in U87MG tumorsphere cells grown in serum-free medium than in U87MG adherent cells cultured in 10% serum-supplemented medium, suggesting that PRMT1 plays a crucial role in GSC growth (Figure 1A). 

To investigate whether the inhibition of PRMT1 activity affects GSC proliferation, furamidine, a specific PRMT1 inhibitor, was used. When U87MG-derived GSCs were treated with various concentrations of furamidine for 7 days, furamidine dose-dependently inhibited the proliferation and tumorsphere formation ability of U87MG GSCs with an IC_50_ value of 15.64 μM (Figure 1B). Notably, the antiproliferative effect of furamidine on U87MG GSCs was stronger than that on U87MG adherent cells and 267B1 normal prostate epithelial cells (Figures S2 and S3). To evaluate the effect of furamidine on the tumorigenic ability of GSCs in vivo, we performed a chick chorioallantoic membrane (CAM) assay in which U87MG-derived GSCs were implanted. As shown in Figure 1C, the tumor weight of the control group was 4.9 mg. In comparison, the tumor weight of the furamidine-treated group was 1.2 mg, indicating that furamidine effectively inhibited GSC-derived tumor growth in vivo. These results suggest that the PRMT1 inhibitor furamidine may have therapeutic potential in inhibiting GSC proliferation both in vitro and in vivo.

### 2.2. Furamidine Induces Cell Cycle Arrest and Apoptosis in U87MG-Derived GSCs

**Figure 2:**
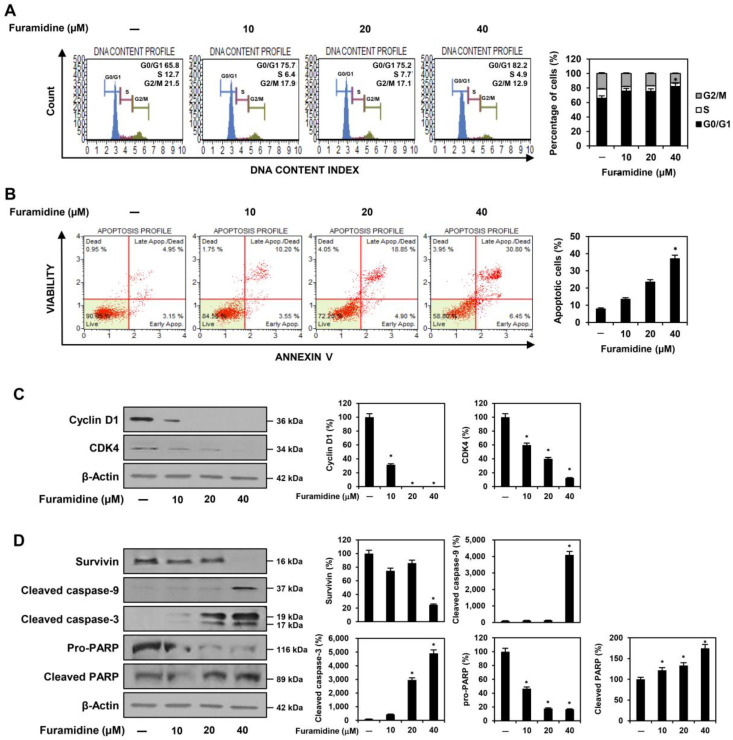
Effect of furamidine on cell cycle and apoptosis in U87MG-derived GSCs. (**A**–**D**) U87MG-derived GSCs were treated with furamidine (10, 20, and 40 μM) for 48 h. (**A**) Cells were stained with Muse^®^ Cell Cycle reagent, and cell cycle distribution was measured using the Muse Cell Analyzer. (**B**) Cells were stained with Muse^®^ Annexin V and Dead Cell reagent, and apoptotic cells were detected using the Muse Cell Analyzer. (**C**,**D**) The expression levels of cell cycle and apoptosis-related proteins were detected by Western blotting and quantified by densitometry. β-Actin levels were used as a loading control. * *p* < 0.05 vs. the control.

Flow cytometry assessed whether furamidine inhibited GSC proliferation by affecting cell cycle arrest and apoptotic cell death. To investigate the effect of furamidine on the cell cycle distribution of U87MG-derived GSCs, the cells were treated with 10, 20, and 40 μM of furamidine for 48 h. Compared with untreated control cells, furamidine treatment significantly increased the cell population in the G0/G1 phase. It decreased the cell population in the S and G2/M phases, indicating that furamidine caused G0/G1 cell cycle arrest in U87MG-derived GSCs (Figure 2A). Next, we examined the effects of furamidine on apoptosis. Furamidine treatment dose-dependently increased the percentage of apoptotic cells in U87MG-derived GSCs (Figure 2B). These data suggest that the anti-proliferative effect of furamidine on GSCs may be related to cell cycle arrest and the induction of apoptosis.

The cell cycle checkpoint proteins cyclin D1 and CDK4 play important roles in the transition from the G0/G1 phase to the S phase of the cell cycle [19,20]. Thus, we determined whether furamidine affects the expression of these cell cycle regulators in U87MG-derived GSCs. Treatment with furamidine significantly decreased the expression of cyclin D1 and CDK4, indicating that furamidine-mediated G0/G1 phase arrest in U87MG GSCs may be associated with the downregulation of cyclin D1/CDK4 (Figure 2C).

Next, we evaluated whether furamidine regulates the mitochondria-mediated apoptotic pathway in U87MG-derived GSCs. Survivin is an anti-apoptotic protein that regulates cell division and inhibits cell death by blocking caspase activation [21]. Caspase-9 and caspase-3 are critical effectors of mitochondria-mediated apoptosis, which is activated by proteolytic cleavage and promotes cell death by cleaving specific substrates, including nuclear poly (ADP-ribose) polymerase (PARP) [22]. As shown in Figure 2D, furamidine treatment upregulated the protein levels of cleaved caspase-9, cleaved caspase-3, and cleaved PARP and downregulated the protein levels of survivin and pro-PARP. These results suggest that furamidine can induce apoptosis in U87MG-derived GSCs by activating the caspase-mediated apoptotic pathway.

**Figure 3:**
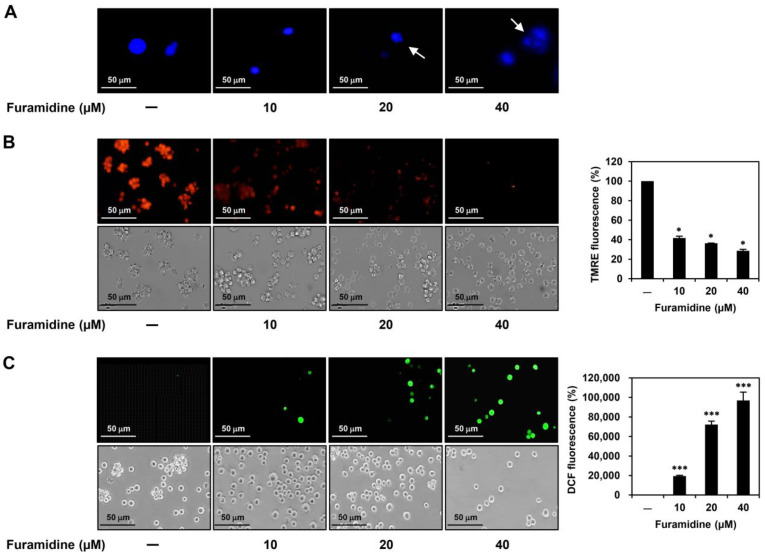
Effect of furamidine on features of mitochondria-mediated apoptosis in U87MG-derived GSCs. (**A**–**C**) U87MG-derived GSCs were treated with furamidine (10, 20, and 40 μM) for 48 h. (**A**) Changes in nuclear morphology were observed by DAPI staining under a fluorescence microscope. Condensed and fragmented nuclei were indicated by white arrows. (**B**) MMP was detected by TMRE staining. Fluorescent images were quantified with densitometry. (**C**) Intracellular ROS levels were detected by DCFH-DA staining. DCF fluorescence was quantified by densitometry. * *p* < 0.05, *** *p* < 0.001 vs. the control.

To further elucidate the proapoptotic effects of furamidine on U87MG-derived GSCs, we investigated several representative features of mitochondria-mediated apoptosis. The 4′,6-diamidino-2-phenylindole (DAPI) staining showed that furamidine treatment caused nuclear condensation and fragmentation in U87MG-derived GSCs (Figure 3A). In addition, staining with tetramethylrhodamine ethyl ester (TMRE), a red-orange fluorescent dye that accumulates in the mitochondria in proportion to mitochondrial membrane potential (MMP), revealed that furamidine treatment led to a significant loss of MMP in U87MG-derived GSCs (Figure 3B). Furthermore, dichlorodihydrofluorescein diacetate (DCFH-DA) staining demonstrated that furamidine markedly increased reactive oxygen species (ROS) production in U87MG-derived GSCs (Figure 3C). Together, these results indicate that the anti-proliferative effect of furamidine in GSCs is related to the activation of mitochondria-mediated intrinsic apoptosis.

### 2.3. Furamidine Inhibits STAT3 Activation and Stemness Marker Expression in U87MG-Derived GSCs

**Figure 4:**
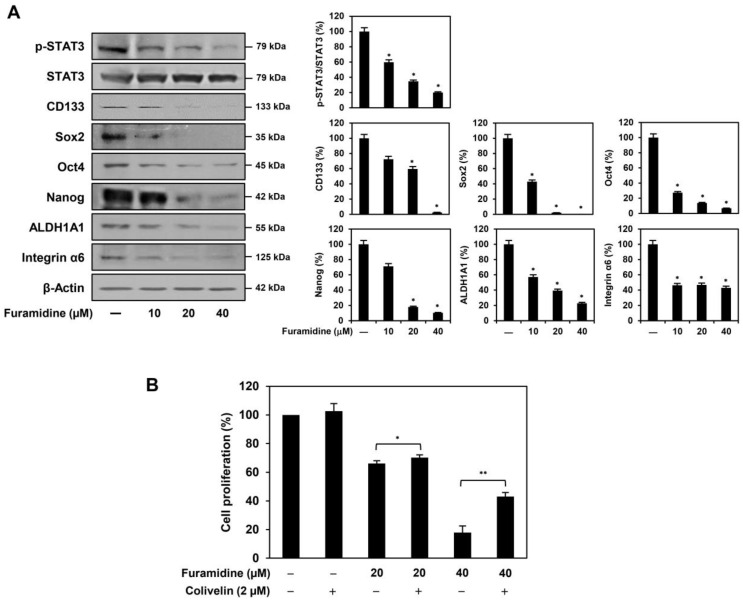
Effect of furamidine on STAT3 and stemness markers in U87MG-derived GSCs. (**A**) U87MG-derived GSCs were treated with furamidine (10, 20, and 40 μM) for 48 h. Protein levels were detected by Western blotting and quantified by densitometry. β-Actin levels were used as a loading control. * *p* < 0.05 vs. the control. (**B**) Effect of colivelin on the antiproliferative activity of furamidine in U87MG-derived GSCs. Cells were incubated for 7 days after treatment with colivelin and furamidine. The CellTiter-Glo^®^ luminescent assay was performed to measure cell proliferation. * *p* < 0.05, ** *p* < 0.005.

STAT3 activation contributes to the proliferation and maintenance of the pluripotency of GSCs by upregulating the expression of a variety of stemness-related genes, including CD133, Sox2, Oct4, Nanog, ALDH1A1, and integrin α6 [23,24]. Therefore, we investigated whether the inhibition of PRMT1 by furamidine affects the activation of STAT3 in U87MG-derived GSCs. As shown in Figure 4A, furamidine treatment significantly reduced the expression of phosphorylated STAT3 without affecting the total STAT3 levels. Consequently, the inhibition of STAT3 activity by furamidine resulted in a significant decrease in the expression of key GSC markers, including CD133, Sox2, Oct4, Nanog, ALDH1A1, and integrin α6 (Figure 4A). In addition, we confirmed whether the antiproliferative effect of furamidine on GSCs was related to the inactivation of STAT3. Colivelin, a selective STAT3 activator, partially rescued the proliferative ability of U87MG-derived GSCs that was inhibited by furamidine (Figure 4B). These results imply that the PRMT1 inhibitor furamidine may suppress the proliferation of U87MG-derived GSCs by inactivating STAT3, a critical regulator of GSC growth and maintenance. 

### 2.4. PRMT1 Knockdown Suppresses Proliferation of U87MG-Derived GSCs Both In Vitro and In Vivo

**Figure 5:**
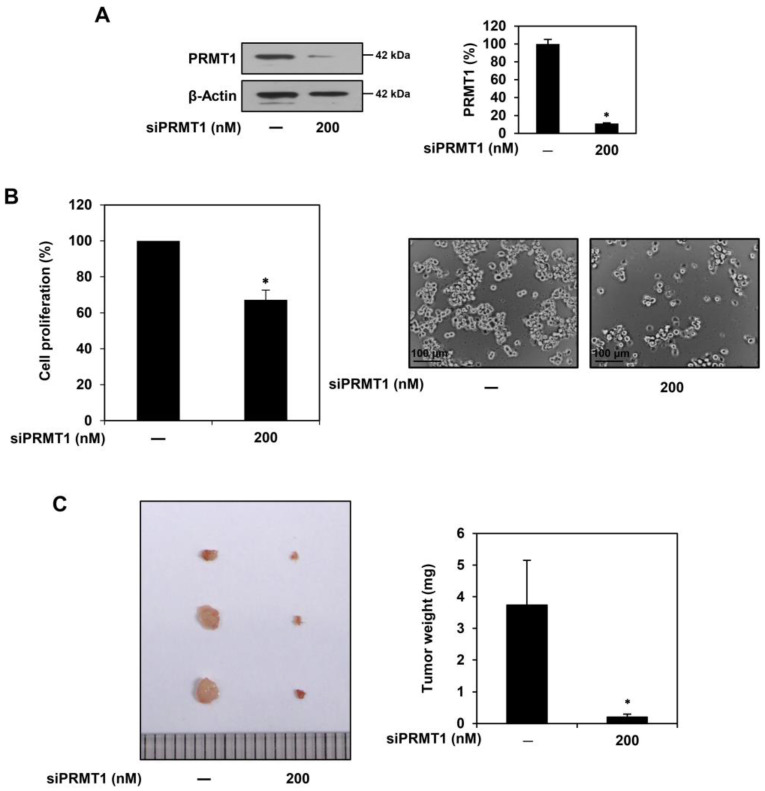
Effect of PRMT1 knockdown on proliferation of U87MG-derived GSCs in vitro and in vivo. (**A**) U87MG-derived GSCs were transfected with either PRMT1 siRNA (200 nM) or negative control siRNA for 48 h. The knockdown of the PRMT1 gene was confirmed by Western blotting. β-Actin levels were used as an internal control. (**B**) Following genetic knockdown, cell proliferation was measured using the CellTiter-Glo^®^ luminescent assay system. (**C**) Effect of PRMT1 knockdown on tumor growth derived by U87MG GSCs in a CAM model. * *p* < 0.05 vs. the control.

To further elucidate whether PRMT1 plays an important role in GSC proliferation, we performed knockdown experiments using siRNAs targeting PRMT1. The U87MG-derived GSCs were transfected with either a PRMT1-specific siRNA (siPRMT1) or a negative control siRNA, and a significant reduction in PRMT1 expression was confirmed by Western blotting (Figure 5A). PRMT1 knockdown effectively suppressed the proliferation of U87MG-derived GSCs (Figure 5B). In addition, the PRMT1 knockdown group (tumor weight of 0.2 mg) showed markedly inhibited in vivo tumor growth of U87MG GSCs compared with the negative control group (tumor weight of 3.7 mg) in the CAM model (Figure 5C). These results suggest that PRMT1 positively regulates the proliferative capacity of GSCs. Therefore, targeting PRMT1 may be a promising strategy by which to eradicate GSCs. 

### 2.5. PRMT1 Knockdown Downregulates GBM Stemness Regulators in U87MG-Derived GSCs

**Figure 6:**
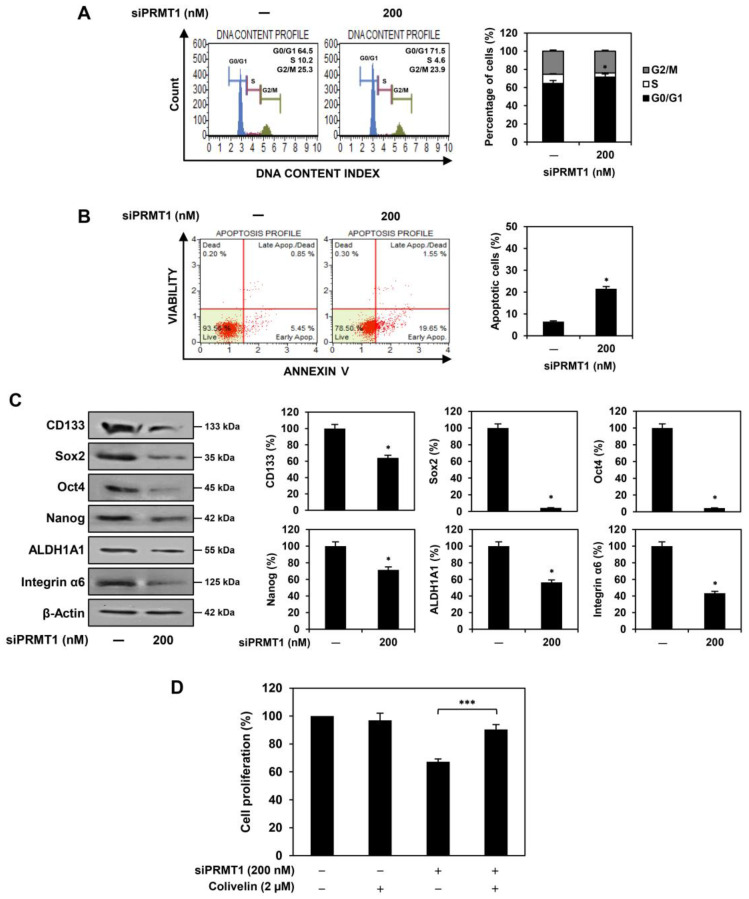
Effect of PRMT1 knockdown on STAT3 and stemness markers in U87MG-derived GSCs. (**A**) Effect of PRMT1 knockdown on the cell cycle. (**B**) Effect of PRMT1 knockdown on apoptotic cell death. (**C**) Effect of PRMT1 knockdown on the protein expression levels of GSC markers. * *p* < 0.05 vs. the control. (**D**) Effect of colivelin on the antiproliferative activity of PRMT1 knockdown in U87MG-derived GSCs. *** *p* < 0.001.

We assessed whether the inhibition of U87MG GSC proliferation by PRMT1 silencing was associated with cell cycle regulation and apoptosis. PRMT1 knockdown increased the G0/G1 phase cell population compared with the control cells (Figure 6A). In addition, PRMT1 knockdown promoted apoptosis compared to the control cells (Figure 6B). These data show that PRMT1 silencing inhibited the proliferation of U87MG-derived GSCs by inducing G0/G1 cell cycle arrest and apoptosis, similar to the effects of furamidine treatment. 

Next, we investigated the effects of PRMT1 knockdown on the expression of key GSC markers. PRMT1 silencing effectively reduced the expression of CD133, Sox2, Oct4, Nanog, ALDH1A1, and integrin α6 in U87MG-derived GSCs (Figure 6C). Moreover, the STAT3 activator colivelin significantly restored the proliferative capacity of U87MG GSCs, which was suppressed by PRMT1 knockdown (Figure 6D). These results demonstrate that PRMT1 may play a key role in activating STAT3 and its downstream GBM stemness regulators, contributing to the growth and maintenance of GSCs. 

### 2.6. Combined Treatment with Furamidine and Berbamine Increases Lethality of U87MG-Derived GSCs

**Figure 7:**
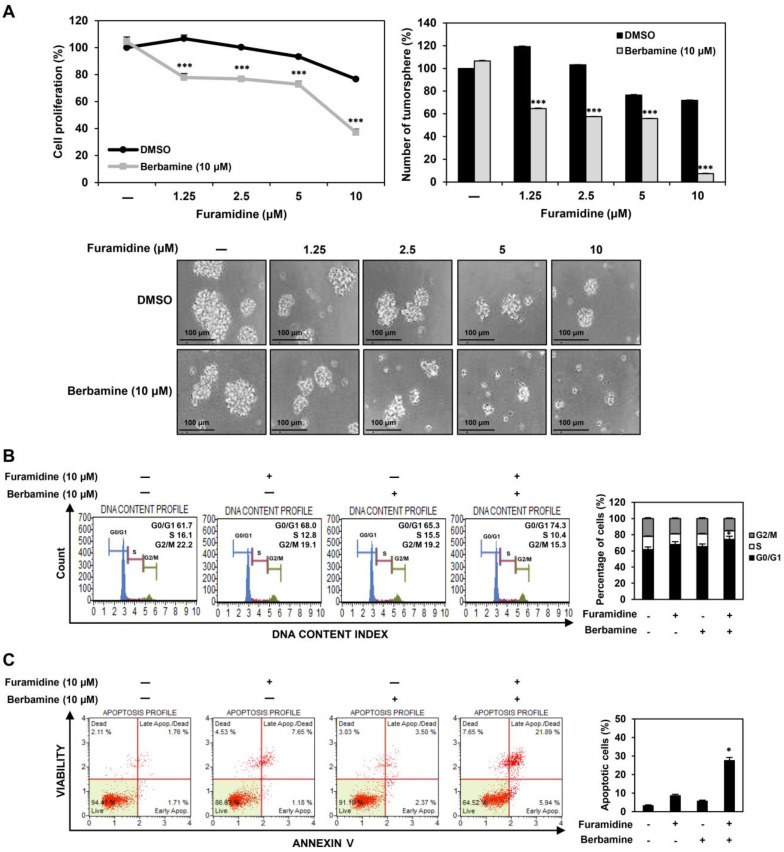
Effect of combined treatment of furamidine and berbamine on proliferation of U87MG-derived GSCs. (**A**) Effect of combined treatment with furamidine and berbamine for 7 days on the proliferation and tumorsphere formation in U87MG-derived GSCs. (**B**,**C**) Effect of combined treatment with furamidine and berbamine for 48 h on (**B**) the cell cycle and (**C**) apoptotic cell death in U87MG-derived GSCs. * *p* < 0.05, *** *p* < 0.001 vs. the compound alone.

Exploring new drug combinations to block the growth of GSCs more effectively is an attractive therapeutic approach to overcoming drug resistance in GBM treatment [25]. In our previous studies, berbamine, a CaMKIIγ inhibitor, potentiated the antiproliferative effects of neurokinin 1 receptor (NK1R) inhibitors, such as SR 140333 and aprepitant, and arcyriaflavin A, a CDK4 inhibitor, on GSCs [26,27,28]. In this study, we evaluated the effects of the combined treatment with furamidine and berbamine on the growth of GSCs. Among the administered doses, the combined treatment of furamidine and berbamine at 10 μM each most effectively inhibited the proliferation and tumorsphere formation of U87MG-derived GSCs compared with treatment with single compounds (Figure 7A). We further investigated whether the combined effect of furamidine and berbamine on the inhibition of GSC proliferation was related to promoting cell cycle arrest and apoptosis. Compared with treatment with single compounds, co-treatment with furamidine and berbamine at 10 μM each more strongly induced cell cycle arrest at the G0/G1 phase and apoptosis in U87MG-derived GSCs (Figure 7B,C). 

**Figure 8:**
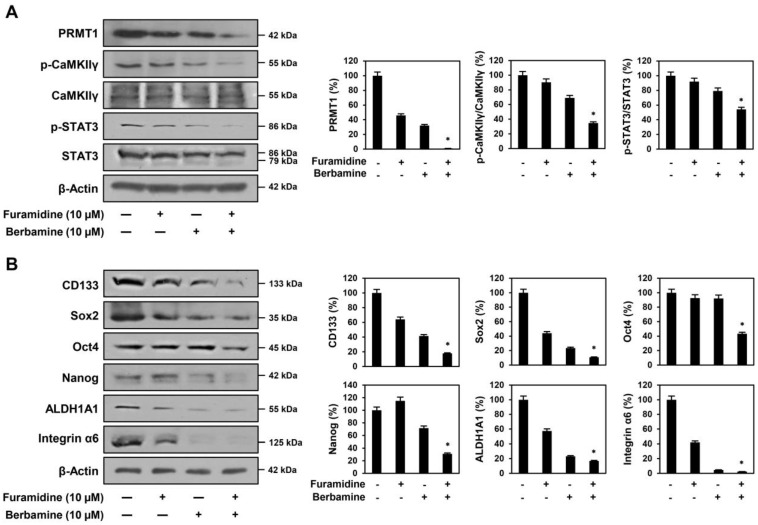
Effect of combined treatment of furamidine and berbamine on (**A**) PRMT1, CaMKIIγ, STAT3, and (**B**) stemness markers in U87MG-derived GSCs. (**A**,**B**) U87MG-derived GSCs were treated with the indicated concentrations of furamidine and berbamine for 48 h. Protein levels were detected by Western blotting and quantified by densitometry. β-Actin levels were used as a loading control. * *p* < 0.05 vs. the compound alone.

Next, we assessed whether this combination effect of the two compounds in U87MG-derived GSCs resulted from the blockade of the functional interaction between PRMT1 and CaMKIIγ. Concurrent treatment of furamidine and berbamine potently suppressed the expression of PRMT1 and phosphorylated CaMKIIγ compared with single-compound treatments (Figure 8A). Moreover, simultaneous treatment with both compounds inhibited the phosphorylation of more STAT3 than treatment with a single compound (Figure 8A). The increased inhibition of STAT3 activity by combined treatment with both compounds resulted in a greater decrease in the expression levels of crucial GSC markers, including CD133, Sox2, Oct4, Nanog, ALDH1A1, and integrin α6 (Figure 8B). Collectively, these results demonstrate that the combination of furamidine and berbamine more effectively suppresses the growth of U87MG-derived GSCs through the increased downregulation of STAT3-mediated downstream GBM stemness regulators by blocking the functional interaction between PRMT1 and CaMKIIγ. 

## 3. Discussion

GSCs are a subpopulation of GBM cells with high self-renewal and multi-lineage differentiation capacities, resulting in tumor initiation and progression, metastasis, resistance to chemotherapy, and rapid tumor recurrence [6,7]. Therefore, the development of potential GSC-targeted therapies may improve treatment outcomes in GBM. Several stemness regulators and signaling pathways that contribute to the growth and maintenance of GSCs have been characterized, and their inhibitors have shown significant therapeutic efficacy in eradicating GSCs [8,9]. Nevertheless, new molecular targets related to the malignant features of GSCs are required to overcome drug resistance and treatment failures.

In this study, we demonstrated for the first time that PRMT1 plays a critical role in promoting GSC growth. Our results showed a significantly increased level of PRMT1 protein expression in U87MG tumorsphere cells grown in serum-free medium compared to U87MG adherent cells cultured in 10% serum-supplemented medium, indicating that PRMT1 may contribute to the stem-like properties of GBM. In addition, the PRMT1 inhibitor, furamidine, potently suppressed the proliferation and tumorsphere formation of U87MG-derived GSCs by inducing G0/G1 cell cycle arrest and apoptosis. The anti-proliferative effect of furamidine on U87MG GSCs was related to the downregulation of cyclin D1/CDK4 and activation of the intrinsic apoptotic pathway, including nuclear condensation and fragmentation, loss of MMP, increased ROS production, and modulation of the survivin/caspase-9/caspase-3/PARP axis. Furthermore, we showed that inhibiting PRMT1 function using siRNA targeting PRMT1 reduced the proliferation of U87MG-derived GSCs by inducing G0/G1 cell cycle arrest and apoptosis, similar to the effects of furamidine treatment. Moreover, furamidine treatment and PRMT1 knockdown strongly suppressed in vivo tumor growth of U87MG GSCs in a CAM model. These results demonstrated the potential of PRMT1 as a novel therapeutic target for disrupting GSC growth. 

Accumulating evidence has shown that STAT3 activation plays an important role in the growth and maintenance of GSCs by increasing the expression of major cancer stemness markers, including CD133, Sox2, Oct4, Nanog, ALDH1A1, and integrin α6 [23,24]. When a cytokine or growth factor binds to a specific receptor, STAT3 is phosphorylated by JAK bound to the receptor and then forms a homodimer or heterodimer [29]. Subsequently, dimerized STAT3 translocates to the nucleus and acts as a transcriptional activator that induces the expression of stemness regulators [30]. Recent studies revealed that PRMT1 directly methylates STAT3 and enhances its activation [31]. As a result, methylation of STAT3 by PRMT1 activates STAT3-mediated signaling pathways, leading to the progression and metastasis of several cancers, such as GBM and hepatocellular carcinoma [31,32,33]. PRMT1 is highly expressed in mouse embryonic neural stem/progenitor cells and promotes astrocyte differentiation by methylating and activating STAT3 [34]. These reports imply that PRMT1 affects the growth of GSCs by regulating STAT3 methylation and activation. Our results showed that furamidine significantly reduced the expression of phosphorylated STAT3 in U87MG-derived GSCs. Accordingly, the inactivation of STAT3 by furamidine reduced the expression of key GSC regulators, including CD133, Sox2, Oct4, Nanog, ALDH1A1, and integrin α6. Additionally, colivelin, a STAT3 activator, partially rescued the proliferative ability of U87MG GSCs, which was inhibited by furamidine. Moreover, PRMT1 knockdown using siRNA showed similar results to furamidine treatment of U87MG-derived GSCs. Therefore, our results suggest that PRMT1 promotes the proliferation of GSCs by activating STAT3 to upregulate the expression of cancer stemness regulators. 

**Figure 9:**
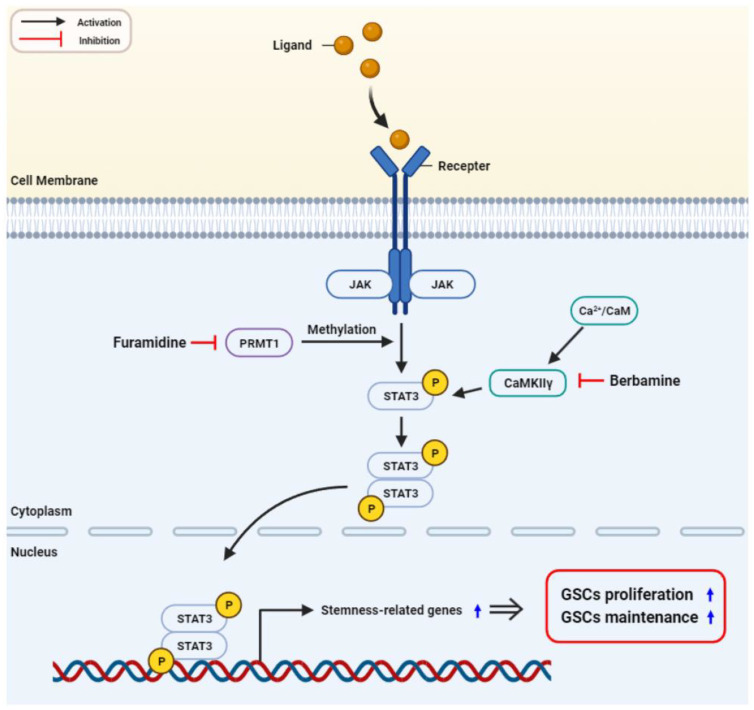
Proposed functional role of PRMT1 in GSC growth. Inhibition of PRMT1 suppresses the growth of GSCs by blocking the STAT3 signaling pathway.

Recently, emerging evidence has revealed that CaMKIIγ, a multifunctional serine/threonine kinase, plays a critical role in maintaining the stem-like features of cancer cells by acting as a molecular switch that regulates several cancer stemness-related signaling pathways, such as NF-κB, Wnt/β-catenin, Notch, and STAT3 [35,36,37]. In addition, CaMKIIγ inhibitors such as berbamine, hydrazinobenzoylcurcumin, and KN93 suppressed the growth of blood, lung, liver, prostate, and brain cancer stem cells (CSCs) [35,36,38,39]. Notably, these CaMKIIγ inhibitors potentiated the antiproliferative effects of NK1R inhibitors, such as SR 140333 and aprepitant, and the CDK4 inhibitor arcyriaflavin A on GSCs both in vitro and in vivo [28]. Thus, CaMKIIγ is a promising anticancer target for combating CSCs, including GSCs. In this study, our results showed that the combined treatment with furamidine and berbamine more strongly inhibited the proliferative and tumorsphere formation abilities of U87MG-derived GSCs by further enhancing G0/G1 cell cycle arrest and apoptosis compared with single compound treatment. We also demonstrated that the increased inhibitory effect on U87MG GSC growth by combined treatment of both compounds was associated with a stronger downregulation of STAT3-mediated downstream GBM stemness regulators by simultaneously blocking PRMT1 and CaMKIIγ activities (Figure 9). Therefore, a combination therapy with furamidine and berbamine may be a new treatment option for patients with GBM. Together, these findings support the potential of PRMT1 and its inhibitor furamidine as a novel therapeutic target and drug candidate for effectively disrupting GSC growth.

## 4. Materials and Methods

### 4.1. Materials

Furamidine (PRMT1 inhibitor), berbamine (CaMKIIγ inhibitor), and colivelin (STAT3 activator) were purchased from Tocris (Bristol, UK), Sigma-Aldrich (St. Louis, MO, USA), and MedChemExpress (Monmouth Junction, NJ, USA), respectively. The compounds were dissolved in dimethyl sulfoxide (DMSO) at a final concentration of 100 mM. Minimum Essential Medium (MEM) and Dulbecco’s Modified Eagle’s Medium/Nutrient Mixture F-12 (DMEM/F-12) were purchased from HyClone (Marlborough, MA, USA). Fetal bovine serum (FBS), B-27 serum-free supplement, L-glutamine, penicillin/streptomycin, and trypsin were purchased from Gibco (Grand Island, NY, USA). Penicillin-streptomycin-amphotericin B and Accutase were purchased from Lonza (Walkersville, MD, USA). EGF and bFGF were purchased from Prospecbio (East Brunswick, NJ, USA). Heparin, extracellular matrix (ECM) gel from Engelbreth-Holm-Swarm murine sarcoma, DAPI, and DCFH-DA were purchased from Sigma-Aldrich (St. Louis, MO, USA). TMRE was purchased from Invitrogen (Carlsbad, CA, USA). CellTiter-Glo^®^ Luminescent Cell Viability Assay kit was purchased from Promega (Madison, WI, USA). Muse^®^ Cell Cycle and Muse^®^ Annexin V and Dead Cell kits were purchased from Luminex (Austin, TX, USA). Antibodies against cyclin D1, survivin, cleaved caspase-9, cleaved caspase-3, PARP, STAT3, phospho-STAT3, CD133, Sox2, Oct4, Nanog, ALDH1A1, integrin α6, rabbit IgG, and mouse IgG were purchased from Cell Signaling Technology (Danvers, MA, USA). Anti-CaMKIIγ and anti-phospho-CaMKIIγ were purchased from Thermo Fisher Scientific (Rockford, IL, USA). Anti-PRMT1 and anti-CDK4 were purchased from Santa Cruz Biotechnology (Dallas, TX, USA). Anti-β-actin was purchased from Abcam (Cambridge, UK).

### 4.2. Cell Culture

U87MG human GBM cell line was purchased from Korean Cell Line Bank (Seoul, Republic of Korea). Adherent cells were grown in MEM containing 10% FBS and 1% penicillin-streptomycin-amphotericin B and subcultured using trypsin. Stem-like tumorsphere cells were cultured in DMEM/F-12 containing 1 × B-27, 5 μg/mL heparin, 2 mM L-glutamine, 20 ng/mL EGF, 20 ng/mL bFGF, and 1% penicillin/streptomycin [17,18]. The tumorsphere cells grown in serum-free medium were subcultured by dissociating with Accutase. All cells were maintained at 37 °C in a humidified 5% CO_2_ incubator (Thermo Scientific, Vantaa, Finland).

### 4.3. Cell Proliferation Assay

U87MG-derived GSCs (3 × 10^3^ cells/well) were seeded in a 96-white-well culture plate and treated with different concentrations of furamidine for 7 days. CellTiter-Glo^®^ Cell Viability Assay reagent (20 μL/well) was added to each well, and luminescence was detected by a microplate reader (BioTek, Inc., Winooski, VT, USA) [40]. IC_50_ value was analyzed using GraphPad Prism 5 (GraphPad Software, La Jolla, CA, USA).

### 4.4. Tumorsphere Formation Assay

U87MG-derived GSCs (3 × 10^3^ cells/well) were seeded in a 96-well culture plate and exposed to different concentrations of furamidine. Following incubation for 7 days, formed tumorspheres (>70 μm in diameter) were observed and counted under an optical microscope (Olympus, Tokyo, Japan) [40].

### 4.5. CAM Assay

The CAM assay has been widely used as a simple, rapid, inexpensive, and naturally immunodeficient in vivo model to evaluate the antiangiogenic, antitumor, delivery, and toxicological effects of drugs [41]. Fertilized chicken eggs were incubated for 6 days in a humidified egg incubator at 37 °C, and a small hole of less than 1 cm was made at the wider end of the egg. U87MG-derived GSCs (15 × 10^5^ cells/egg), furamidine (10 µg), and ECM gel (10 mg/mL, 40 µL/egg) were mixed and solidified in an incubator (37 °C, 5% CO_2_) for 1 h. The mixture was placed on CAM. After 7 days of incubation, the tumors formed on CAM were harvested and weighed [40].

### 4.6. Cell Cycle Analysis

U87MG-derived GSCs (2 × 10^5^ cells/well) were seeded in a 60-mm culture dish and treated with furamidine for 48 h. Cells were collected, washed with PBS, fixed with cold 70% ethanol for 3 h, and stained with 200 µL of Muse^®^ Cell Cycle reagent for 30 min [40]. Cell cycle distribution of stained cells was analyzed using the Guava^®^ Muse^®^ Cell Analyzer (MuseSoft_V1.8.0.3; Luminex, Austin, TX, USA).

### 4.7. Apoptosis Analysis

U87MG-derived GSCs (2 × 10^5^ cells/well) were seeded in a 60-mm culture dish and treated with furamidine for 48 h. Cells were collected and stained with 100 µL of Muse^®^ Annexin V and Dead Cell reagent for 30 min [40]. The percentage of apoptotic cells was analyzed using the Guava^®^ Muse^®^ Cell Analyzer (MuseSoft_V1.8.0.3; Luminex, Austin, TX, USA). 

### 4.8. Nuclear Fluorescent Staining with DAPI

U87MG-derived GSCs (1 × 10^5^ cells/well) were seeded in a 24-well culture plate and exposed to furamidine for 48 h. Cells were stained with 10 μg/mL of DAPI for 30 min, and nuclear morphology was observed under a fluorescence microscope (Optinity KI-2000F, Korea Lab Tech, Seong Nam, Republic of Korea) [40].

### 4.9. Measurement of MMP

U87MG-derived GSCs (1 × 10^5^ cells/well) were seeded in a 24-well culture plate and exposed to furamidine for 48 h. Cells were stained with 100 nM of TMRE for 20 min, and images were obtained using a fluorescence microscope (Optinity KI-2000F, Korea Lab Tech, Seong Nam, Republic of Korea) [42]. The TMRE fluorescence density was analyzed using the ImageJ 1.5 software (NIH, Bethesda, MD, USA).

### 4.10. Measurement of Intracellular ROS

U87MG-derived GSCs (1 × 10^5^ cells/well) were seeded in a 24-well culture plate and exposed to furamidine for 48 h. Cells were stained with 10 μM of DCFH-DA for 20 min, the levels of intracellular ROS were observed under a fluorescence microscope (Optinity KI-2000F, Korea Lab Tech, Seong Nam, Republic of Korea) [40]. The DCF fluorescence intensity was analyzed using the ImageJ 1.5 software (NIH, Bethesda, MD, USA).

### 4.11. PRMT1-Directed RNA Interference

To knockdown the expression of the PRMT1 gene, a PRMT1-specific siRNA was obtained from Bioneer (Daejeon, Republic of Korea). The sense and antisense sequences of PRMT1 siRNA were 5′-GACUUCACCAUCGACCUGGACUUCA-3′ and 5′-UGAAGUCCAGGUCGAUGGUGAAGUC-3′, respectively. Non-targeting scrambled siRNA (Santa Cruz Biotechnology, Dallas, TX, USA) was used as a negative control. Cells were transfected with siRNAs using Lipofectamine^TM^ 2000 Reagent (Invitrogen, NY, USA). The expression levels of PRMT1 were determined by Western blot [28]. 

### 4.12. Western Blot 

Following treatment, cells were collected and lysed with RIPA lysis buffer (ATTO, Tokyo, Japan). Equal amounts of lysates were separated by 10% sodium dodecyl sulfate-polyacrylamide gel electrophoresis and then transferred to polyvinylidene difluoride membranes (Millipore, Hayward, CA, USA). Blots were blocked in 5% skim milk for 1 h and then incubated with primary antibodies (dilution 1:2000–1:10,000) overnight at 4 °C. After incubation with horseradish peroxidase-conjugated secondary antibodies (dilution 1:3000) for 1 h, immunolabeling was detected using an enhanced chemiluminescence kit (Bio-Rad Laboratories, Hercules, CA, USA) [40]. Band density was analyzed using the ImageJ 1.5 software (NIH, Bethesda, MD, USA).

### 4.13. Statistical Analysis

The results were presented as mean ± standard deviation (SD), and statistical analyses were carried out using the SPSS statistics package (SPSS 9.0; Chicago, IL, USA). A one-way analysis of variance (ANOVA) followed by Tukey’s test as post hoc analysis was used to compare the different groups [40]. A *p*-value of <0.05 was considered to be a statistically significant difference and indicated by an asterisk (*). 

## 5. Conclusions

In this study, we first identified the functional role of PRMT1 in GSC growth using the PRMT1 inhibitor, furamidine, and PRMT1-specific siRNA. Furamidine treatment and PRMT1 knockdown significantly suppressed the proliferation and tumorsphere formation of U87MG-derived GSCs by inducing G0/G1 cell cycle arrest and activating the intrinsic apoptotic pathway. Moreover, furamidine treatment and PRMT1 knockdown potently inhibited the in vivo tumor growth of U87MG GSCs in a CAM model. Notably, the antiproliferative effect on GSCs inhibited STAT3 activity and downstream GBM stemness regulators, including CD133, Sox2, Oct4, Nanog, ALDH1A1, and integrin α6. In addition, combined treatment with furamidine and berbamine, a CaMKIIγ inhibitor, more effectively suppressed the growth of U87MG GSCs through the stronger downregulation of STAT3-mediated downstream GBM stemness regulators by simultaneously blocking PRMT1 and CaMKIIγ functions. These findings suggest a new therapeutic approach targeting PRMT1 to obstruct GSC growth.

## Data Availability

Data are contained within the article and Supplementary Materials.

## Supplementary Materials

The following supporting information can be downloaded at: https://www.mdpi.com/article/10.3390/ijms25052950/s1.
