# Supplementary material for: Inhibition of PRMT1 Suppresses the Growth of U87MG-Derived Glioblastoma Stem Cells by Blocking the STAT3 Signaling Pathway
Source: Int J Mol Sci. 2024 Mar 3;25(5):2950. doi: 10.3390/ijms25052950 (PMC10931587; doi:10.3390/ijms25052950)
Supplement: Supplementary file 1 [file ijms-25-02950-s001.zip › ijms-2869342-supplementary.pdf]

Article

Inhibition of PRMT1 Suppresses the Growth of U87MG-Derived Glioblastoma Stem Cells by Blocking the STAT3 Signaling Pathway

Nayeong Yuk [1] and Hye Jin Jung [1,2,3,*]

1 Department of Life Science and Biochemical Engineering, Graduate School, Sun Moon University, Asan 31460, Republic of Korea; nayeong7249@sunmoon.ac.kr
2 Department of Pharmaceutical Engineering and Biotechnology, Sun Moon University, Asan 31460, Republic of Korea
3 Genome-Based BioIT Convergence Institute, Sun Moon University, Asan 31460, Republic of Korea
* Correspondence: poka96@sunmoon.ac.kr; Tel.: +82-41-530-2354; Fax: +82-41-530-2939

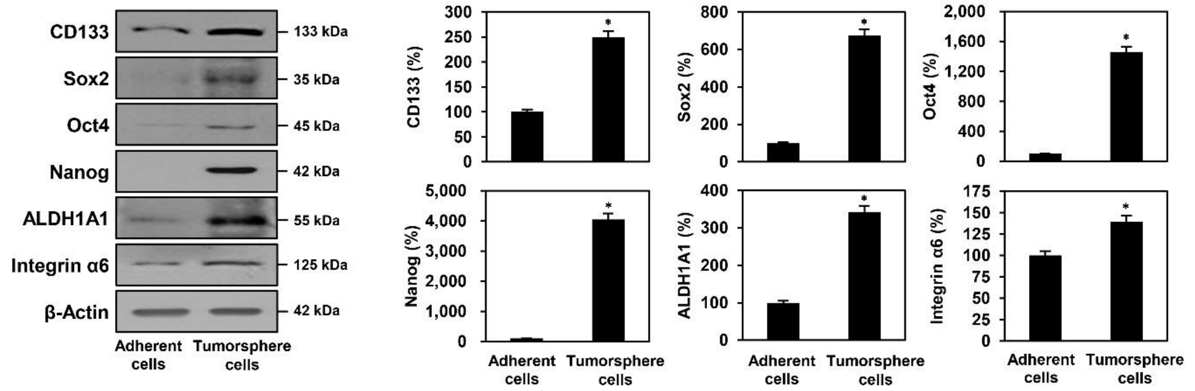

Figure S1. Protein expression levels of key GSC markers in U87MG adherent and tumorsphere cells. Protein levels were detected by western blotting and quantified by densitometry. β-Actin levels were used as a loading control. * $p < 0.05$ vs. the adherent cells.

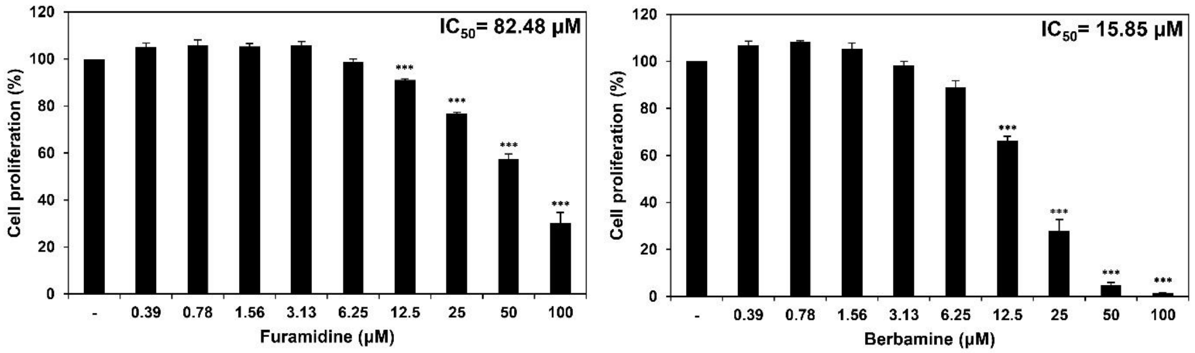

Figure S2. Effects of furamidine and berbamine on the proliferation of U87MG adherent cells. Cells were treated with furamidine or berbamine at various concentrations (0–100 μM) for 72 h. Cell proliferation was measured using the CellTiter-Glo® luminescent assay system. *** $p < 0.001$ vs. the control.

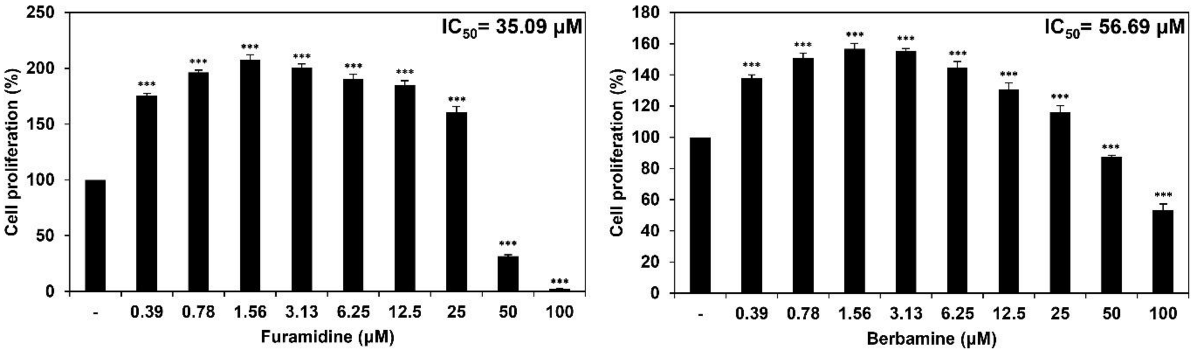

Figure S3. Effects of furamidine and berbamine on the proliferation of 267B1 normal prostate epithelial cells. Cells were treated with furamidine or berbamine at various concentrations (0–100 μM) for 72 h. Cell proliferation was measured using the CellTiter-Glo® luminescent assay system. *** $p < 0.001$ vs. the control.
